# Manure substitution counteracts mineral nitrogen-driven erosion of the *phoD* guild and supports phosphorus mobilization in acidified paddy soil

**DOI:** 10.1093/ismeco/ycag261

**Published:** 2026-09-10

**Authors:** Jitao Huang, Bangxiao Zheng, Yuxuan Mo, Salimzoda Amonullo, Weiyi Lv, Xianyong Lin, Qing-Fang Bi

**Affiliations:** Center for Ecology & Health Innovative Research, Xiamen University of Technology, Xiamen 361024, P. R. China; Fenner School of Environment & Society, The Australian National University, Canberra, Australian Capital Territory 2601, Australia; Center for Ecology & Health Innovative Research, Xiamen University of Technology, Xiamen 361024, P. R. China; Xiamen Key Laboratory of Membrane Research and Application, Xiamen University of Technology, Xiamen 361024, P. R. China; Global Ecology Unit, CREAF-CSIS-UAB, Bellaterra 08193, Spain; Faculty of Biological and Environmental Sciences, Ecosystems and Environment Research Programme, University of Helsinki, Niemenkatu 73, Lahti 15140, Finland; Global Ecology Unit, CREAF-CSIS-UAB, Bellaterra 08193, Spain; Yunnan Key Laboratory of Forest Ecosystem Stability and Global Change, Xishuangbanna Tropical Botanical Garden, Chinese Academy of Sciences, Mengla, Yunnan 666303, P. R. China; Institute of Soil Sciences and Agrochemistry, Tajik Academy of Agricultural Sciences, Dushanbe 734025, Tajikistan; Center for Ecology & Health Innovative Research, Xiamen University of Technology, Xiamen 361024, P. R. China; MOE Key Laboratory of Environment Remediation and Ecological Health, College of Environmental and Resource Sciences, Zhejiang University, Hangzhou 310058, P. R. China; Department Biogeochemical Integration, Max Planck Institute for Biogeochemistry, Jena 07745, Thuringia, Germany; Institute of Agricultural and Nutritional Sciences, Soil Biogeochemistry, Martin Luther University Halle-Wittenberg, Halle (Saale) 06120, Saxony-Anhalt, Germany; German Centre for Integrative Biodiversity Research (iDiv) Halle-Jena-Leipzig, Leipzig D-04103, Saxony, Germany

**Keywords:** *phoD*-harbouring bacteria, *phoD* gene abundance, alkaline phosphatase, soil pH buffering, manure substitution, microbial phosphorus mobilization

## Abstract

Maintaining microbially mediated phosphorus mobilization under intensive nitrogen fertilization is difficult in acidified paddy soils. Using a decade-long field experiment in a rice–oilseed rape rotation, we compared an ordered manure-substitution series, in which manure input increased while mineral nitrogen input decreased, with a mineral nitrogen dose gradient. Increasing mineral nitrogen input was associated with lower diversity and absolute abundance of the *phoD*-harbouring bacterial guild and a marked shift in community composition. Across the manure-substitution series, soil pH increased from 5.94 to 6.62, potential alkaline phosphatase activity increased by up to 158%, and Olsen-extractable phosphorus increased by up to 182%. The diversity, composition, and absolute abundance of the *phoD* guild changed progressively across the same series. Distance-based redundancy analysis identified soil pH as the strongest measured marginal correlate of community composition, whereas variation partitioning indicated that the effects of pH, carbon, and phosphorus were largely shared. An exploratory structural equation model was consistent with conditional associations of both community composition and absolute gene abundance with alkaline phosphatase activity, which was, in turn, associated with Olsen-extractable phosphorus. The highest observed means for gene abundance, grain yield, and grain phosphorus removal occurred in the intermediate manure treatment, whereas treatment-level copper and zinc concentrations increased towards the highest-manure treatment. Together, these findings indicate that manure substitution can counteract mineral nitrogen-associated deterioration of the *phoD* guild and support microbial phosphorus mobilization, although the agronomic and metal responses require confirmation across sites and seasons.

## Introduction

Intensive nitrogen (N) fertilization has supported high crop productivity but has also imposed persistent physicochemical stress on agricultural soils [1]. Global agricultural use of inorganic fertilizers reached ~185 million tonnes in 2022, of which N accounted for 58% [2]. In intensively managed croplands, sustained mineral N input can accelerate soil acidification through proton production during nitrification, thereby altering nutrient availability and constraining microbial activity [3, 4]. Phosphorus (P) cycling is particularly vulnerable because declining pH can increase P retention by iron and aluminium minerals while constraining biological processes that mineralize soil organic P [5]. Chronic N enrichment can also increase ecosystem P demand and alter microbial nutrient allocation [6–9]. Maintaining microbially mediated P mobilization is therefore important for fertility management in acidified agroecosystems [10].

Soil microorganisms contribute to P acquisition by mineralizing organic P compounds and transforming sparingly available P pools [10]. Alkaline phosphatase activity (AKP) is a major component of extracellular phosphomonoester mineralization, and *phoD* is among the most widespread bacterial genes encoding alkaline phosphatase in soils [11, 12]. The diversity and composition of *phoD*-harbouring bacterial communities can influence which populations contribute to P mineralization under a given soil environment, whereas absolute *phoD* gene abundance describes the size of the genetic reservoir potentially contributing to enzyme production [13, 14]. Gene copy number does not directly represent transcription or realized enzyme synthesis, but integrating community composition with quantitative polymerase chain reaction (qPCR)–based abundance provides information that relative sequence profiles alone cannot supply [15]. Recent studies show that long-term organic fertilization can restructure *phoD*-harbouring communities and strengthen associations among *phoD* taxa, phosphatase activity, and crop P acquisition [16–18].

Most fertilization studies have emphasized treatment centroids or changes in dominant taxa, leaving three related uncertainties. First, manure simultaneously modifies soil pH, organic carbon (C), and N and P pools; a response along a manure-substitution series therefore cannot be attributed to N source alone [19, 20]. Second, relative community profiles do not reveal whether a compositional shift is accompanied by expansion or contraction of the functional guild [15]. Third, fertilization may alter among-replicate compositional variability, as well as mean community composition [21]. Dispersion measured at one sampling time can describe spatial heterogeneity among replicate plots, but it does not by itself identify assembly mechanisms or demonstrate temporal stability, resistance, resilience, or functional reliability [21, 22].

Manure substitution may counteract mineral N-associated acidification through several nonexclusive processes. Lower mineral N input can reduce further proton production, while manure can increase buffering capacity and supply organic C and nutrients [23, 24]. These coupled changes may expand the environmental niche available to *phoD*-harbouring bacteria and support AKP-associated P mobilization. However, manure can also introduce agronomic and environmental trade-offs. Crop responses may show diminishing returns as manure input increases [25], while repeated swine-manure application can increase soil copper (Cu) and zinc (Zn) concentrations [26, 27]. A defensible interpretation therefore requires the microbial response to be considered together with soil chemistry, potential enzyme activity, crop performance, and possible environmental costs.

Here, we used a decade-long rice–oilseed rape fertilization experiment containing two complementary contrasts: a mineral N dose gradient spanning low to excessive inputs and an ordered manure-substitution series in which manure input increased while mineral N input decreased. The second contrast did not factorially separate N source and quantity; treatment codes were analysed as ordered levels rather than verified manure-N percentages. We quantified *phoD* diversity, community composition, putative genus-level responses, and absolute gene abundance together with soil C, N, and P properties and phosphatase activities. Archived crop and soil-metal measurements were used as contextual evidence. We hypothesized that: (i) the highest mineral N inputs would be associated with lower *phoD* diversity and absolute abundance; (ii) increasing manure input would be associated with reduced soil acidity, expansion and restructuring of the *phoD* guild, and higher AKP and Olsen-P; and (iii) microbial community structure and absolute *phoD* abundance would retain associations with AKP after correlated soil properties were considered.

## Materials and methods

### Site description and experimental design

A long-term fertilization experiment under a rice–oilseed rape rotation was established in 2008 at the Yangdu Experimental Station, Haining, Zhejiang Province, China (30°26′ N, 120°25′ E). The subtropical monsoon site has a mean annual temperature of 15.9°C and precipitation of 1187 mm. The Gleyi-Stagnic Anthrosol developed from alluvial deposits [28]. Baseline properties of the 0–20 cm layer were pH 6.8, soil organic C 18.5 g kg^−1^, total N 1.85 g kg^−1^, total P 0.62 g kg^−1^, and Olsen-P 12.3 mg kg^−1^.

The randomized complete block experiment had three 10 m × 5 m plots per treatment separated by 1.5 m buffers. The ordered manure-substitution series comprised N4, N3M1, N2M2, and N1M3. During the rice season, mineral N declined from 225 to 180, 150, and 120 kg N ha^−1^ season^−1^, respectively, while fresh composted swine manure increased from 0 to 1500, 3000, and 4500 kg ha^−1^ season^−1^. These categorical codes were not treated as verified manure-N percentages. The mineral N dose series N0, N1, N2, N4, N5, and N6 received 90, 120, 150, 225, 270, and 360 kg N ha^−1^ season^−1^, respectively, during the rice season without manure; N0 was the lowest fertilized treatment, not an unfertilized control. N4 was the shared mineral-fertilizer reference. N4M2 received the N4 mineral N rate plus 3000 kg fresh manure ha^−1^ season^−1^; it was excluded from ordered-series regressions but retained in all-treatment analyses (Table 1).

**Table 1:** Treatment structure and season-specific fertilizer inputs in the long-term rice–oilseed rape rotation experiment.

Treatment set	Treatment	Composted swine manure (kg fresh weight ha ^−1^ season ^−1^ )	Mineral-fertilizer input (kg nutrient ha ^−1^ season ^−1^ )		
			N	P_2_O_5_	K _2_ O
**Rice season**					
Ordered manure substitution	**N4**	0	225	75	90
	**N3M1**	1500	180	75	90
	**N2M2**	3000	150	75	90
	**N1M3**	4500	120	75	90
*Organic addition*	**N4M2**	3000	225	75	90
Mineral N dose gradient	**N0**	0	90	75	90
	**N1**	0	120	75	90
	**N2**	0	150	75	90
	**N5**	0	270	75	90
	**N6**	0	360	75	90
**Oilseed rape season**					
Ordered manure substitution	**N4**	0	150	75	90
	**N3M1**	1500	120	75	90
	**N2M2**	3000	90	75	90
	**N1M3**	4500	60	75	90
*Organic addition*	**N4M2**	3000	150	75	90
Mineral N dose gradient	**N0**	0	90	75	90
	**N1**	0	60	75	90
	**N2**	0	90	75	90
	**N5**	0	180	75	90
	**N6**	0	210	75	90

*Notes:* The three treatment contrasts shared N4 as the mineral-fertilizer reference. N4, N3M1, N2M2, and N1M3 form an ordered treatment series with increasing manure input and decreasing mineral N input; the treatment codes are categorical and were not interpreted as verified manure-N substitution percentages. N4M2 received the N4 mineral N rate plus the manure rate used in N2M2. The mineral N dose gradient comprised N0, N1, N2, N4, N5, and N6 without manure; N4 is listed once within each season because it is shared, and N0 is the lowest mineral N treatment rather than a zero-N control. Manure rates are reported on a fresh-weight basis, whereas N, P_2_O_5_, and K_2_O are mineral-fertilizer nutrient inputs per growing season. Mineral-fertilizer P and K inputs were held constant within each season.

Mineral N, P, and potassium (K) were supplied as urea, single superphosphate, and potassium chloride, respectively, at 75 kg P_2_O_5_ and 90 kg K_2_O ha^−1^ season^−1^. On a dry-weight basis, the composted swine manure contained 18.5 g total N, 12.3 g total P, 9.8 g total K, and 420 g organic matter kg^−1^; its moisture content was 28.4%, pH was 7.62, and the C:N ratio was 18.7. Cu, Zn, cadmium, and lead concentrations were 138, 612, 0.64, and 12.6 mg kg^−1^ dry manure, respectively. Fresh manure was incorporated before crop establishment.

Rice (*Oryza sativa* L., cv. Xiushui 134) was transplanted in late June at ~25 hills m^−2^ and harvested in late October; oilseed rape (*Brassica napus* L.) was sown in late October and harvested in mid-May. Rice fields were maintained under 3–5 cm of water until ~2 weeks before harvest. Weeds were controlled manually, and no pesticides were applied. Aboveground residues were removed, whereas belowground residues and rhizodeposits remained.

Soils were sampled in January 2018 during the oilseed rape seedling stage after 10 years of treatment. Five random 0–20-cm cores per plot were composited, and visible residues, roots, and stones were removed. Field-moist soil was stored at 4°C for no longer than 48 h before enzyme assays, physicochemical subsamples were air-dried, and molecular subsamples were stored at −80°C.

### Soil physicochemical analyses

Air-dried soil was sieved to 2 mm. Soil pH was measured in a 1:2.5 soil-to-water suspension. Total organic carbon (TOC) and total nitrogen (TN) were measured by dry combustion (Vario EL III, Elementar, Germany). Total phosphorus (TP) was determined after H_2_SO_4_–HClO_4_ digestion (5,1, v/v) by molybdenum-blue colorimetry [29]. Olsen-extractable phosphorus (Olsen-P) was extracted with 0.5 M NaHCO_3_ at pH 8.5 and measured at 880 nm [30]. Organic phosphorus (P_o_) was determined by ignition [31] and treated as an operational pool; one analytically impossible negative N4 value was set to missing. Ammonium nitrogen (NH_4_^+^–N) and nitrate nitrogen (NO_3_^−^–N) were extracted from field-moist soil with 2 M KCl and quantified by continuous-flow analysis. Values were expressed on an oven-dry-soil basis.

A principal-component soil N index (PCN) summarized *z*-standardized TN, NH_4_^+^–N, and NO_3_^−^–N. The first component fitted to the 12 N4, N3M1, N2M2, and N1M3 samples explained 51.53% of variance and had loadings of 0.690, 0.673, and −0.267, respectively (Table S1). It was oriented towards higher TN and NH_4_^+^–N and lower NO_3_^−^–N. The fitted centre, scale, and loadings were applied unchanged to all other samples.

### Phosphatase activity assays

Alkaline phosphatase activity (AKP; EC 3.1.3.1), acid phosphatase activity (ACP; EC 3.1.3.2), and phosphodiesterase activity (PDE; EC 3.1.4.1) were measured, following Tabatabai [32]. One gram of field-moist soil was incubated at 37°C for 1 h with 0.05 M *p*-nitrophenyl phosphate for AKP and ACP or bis-*p*-nitrophenyl phosphate for PDE in modified universal buffer at pH 11.0, 6.5, or 8.0, respectively. After termination with 0.5 M NaOH and CaCl_2_, released *p*-nitrophenol was measured at 405 nm after substrate-free correction. Activities were expressed as mg *p*-nitrophenol kg^−1^ dry soil h^−1^. Analytical duplicates were averaged, and inference used three independent plots.

### Agronomic records and contextual Cu/Zn data

Plot-level rice grain yield and grain P concentration for the 2018 rice harvest were obtained from archived records of the same long-term experiment. Apparent grain P removal was calculated as grain yield (kg ha^−1^) × grain P concentration (g kg^−1^)/1000 and expressed as kg P ha^−1^. Because the calculation used grain yield rather than total aboveground biomass, the variable was interpreted as grain P removal rather than total plant P uptake. These agronomic variables were used as contextual responses and were not included as endogenous variables in the structural equation model.

Archived treatment-level mean concentrations of soil Cu and Zn were available for N0, N4, N3M1, N2M2, N1M3, and N4M2. The underlying replicate-level measurements and complete analytical quality-control records were unavailable in the dataset used here. Cu and Zn were therefore displayed only as contextual indicators of potential long-term accumulation, without error bars, hypothesis tests, or inclusion in correlation, regression, or structural equation analyses.

### DNA extraction, phoD amplification, sequencing, and qPCR

DNA was extracted from 0.5 g soil using the FastDNA SPIN Kit for Soil (MP Biomedicals, USA). Integrity was checked by agarose-gel electrophoresis and concentration and purity by NanoDrop spectrophotometry.

An ~350-bp *phoD* fragment was amplified with ALPS-F733 (5′-TGGGAYGATCAYGARGT-3′) and ALPS-R1083 (5′-CTGSGCSAKSACRTTCCA-3′) [33]. Each 25 μl reaction contained 12.5 μl 2× Taq Master Mix, 1 μl of each 10 μM primer, and ~20 ng DNA. Cycling was 95°C for 5 min; 35 cycles of 95°C for 30 s, 55°C for 30 s, and 72°C for 45 s; and 72°C for 10 min. Purified equimolar amplicons were sequenced on an Illumina MiSeq (2 × 250 bp; Majorbio, Shanghai, China).

Absolute *phoD* abundance was measured by qPCR with the same primers on a Bio-Rad CFX96. Plasmid standards covered 10^2^–10^8^ copies μl^−1^. Each 20 μl reaction contained 10 μl 2× SYBR Green master mix, 0.5 μM of each primer, and ~10 ng DNA. Cycling was 95°C for 30 s, followed by 40 cycles of 95°C for 5 s, 55°C for 30 s, and 72°C for 30 s; specificity was checked by a 65°C–95°C melting curve. Efficiencies were 92%–100% and standard-curve *R*^2^ > 0.99. Technical triplicates, no-template controls, and dry-soil normalization were used.

Reads were processed in QIIME 2 v2024.2 using DADA2 for quality filtering, denoising, merging, and chimera removal [34, 35]. Samples were rarefied to 10 483 reads with seed 20 260 721 for α- and β-diversity analyses; nonrarefied counts were converted to relative abundances for genus-level analyses.

### Taxonomic annotation of phoD ASVs

A custom *phoD* protein database was retrieved from UniProtKB on 22 September 2025 using gene_exact:phoD AND taxonomy_id:2. Fragmentary entries were removed and proteins of 350–800 amino acids retained (Data S1).

ASVs were translated, frame-corrected, and aligned to the database with FrameBot v1.1 under OpenJDK 11.0.27 using translation table 11, a 0.40 protein-identity threshold, and BLOSUM62 [36]. The best UniProt accession per ASV was mapped to the OS organism field and compiled as a QIIME 2 taxonomy table.

Because the fragment was short and the identity threshold was 40%, assignments were restricted to putative genera. Genus relative abundance was multiplied by total *phoD* abundance to obtain a qPCR-scaled index, not direct taxon-specific abundance, because qPCR was not taxon specific and gene copy number may vary. The feature and taxonomy tables are Data S2; the full workflow is in the Supplementary Methods.

### Statistical analyses

Statistical analyses were conducted in R v4.3.1 [37] unless otherwise stated. Analyses of the mineral N dose series used 18 samples from N0, N1, N2, N4, N5, and N6; analyses of the ordered manure-substitution series used 12 samples from N4, N3M1, N2M2, and N1M3. Soil-property comparisons additionally included N0 and used 15 samples. Integrated correlations and structural equation modelling used all 30 samples, with the shared N4 samples included once. Treatment effects on soil properties, enzyme activities, α-diversity indices, grain yield, and grain P removal were evaluated by randomized-block analysis of variance, with treatment and block as explanatory terms. Residual normality and variance homogeneity were assessed using Shapiro–Wilk and Levene tests. Log_10_ or square-root transformations were applied where required; if assumptions remained violated, Kruskal–Wallis tests followed by Dunn comparisons were used. *Post hoc* comparisons were conducted only after a significant overall treatment effect, and multiple-comparison *P*-values were adjusted within each response.

For the mineral N dose series, Shannon diversity was related to measured mineral N input using ordinary least-squares linear regression. PCoA1 scores and absolute *phoD* abundance were analysed using second-order polynomial regressions containing linear and quadratic mineral N terms. These models summarized responses across the six fixed rates and were not used to infer ecological thresholds or optimized N rates. For N4, N3M1, N2M2, and N1M3, Shannon diversity, Pielou’s evenness, Chao1 richness, and absolute *phoD* abundance were related to an ordered treatment score of 0, 1, 2, and 3. This score represented increasing manure input and decreasing mineral N input and did not assume verified manure-N percentages. Standardized slopes were reported with 5000-resample bootstrap confidence intervals.

Community analyses used Bray–Curtis dissimilarities calculated from rarefied ASV relative-abundance profiles. Principal coordinates analysis (PCoA) was used for visualization. Treatment centroids were tested by permutational multivariate analysis of variance (PERMANOVA) using adonis2 in vegan v2.6-4 [38] with 9999 block-constrained permutations; multivariate dispersion was assessed using betadisper and permutest. Within-treatment compositional variability was also summarized descriptively using FAVA [21]. Because the manure-series PERMDISP test was not significant and FAVA was calculated from three replicates per treatment, FAVA was not interpreted as evidence of temporal stability or a particular assembly mechanism. Putative genera occurring in at least 25% of samples with an overall mean relative abundance of at least 0.05% were retained. Spearman correlations identified genera associated with the ordered treatment score and qPCR-scaled genus indices associated with AKP; Benjamini–Hochberg false-discovery-rate correction was applied within each test family.

Within the ordered manure-substitution series, associations between community composition and soil pH, TOC, and PCN were examined by distance-based redundancy analysis fitted to Bray–Curtis dissimilarities using capscale in vegan. Overall and marginal effects were evaluated with 9999 block-constrained permutations. Collinearity was examined using pairwise correlations and variance inflation factors. Partial *R*^2^ was calculated as the change in adjusted *R*^2^ after removal of a predictor; negative adjusted or partial values were retained. The conditional association between community structure and AKP was evaluated by multiple regression of standardized AKP on standardized PCoA1, pH, TOC, and PCN. Variation partitioning summarized unique and shared fractions of AKP variation associated with pH, TOC, and TP and was interpreted descriptively because the predictors were correlated and *n* = 12.

Across all 30 samples, Spearman correlations were used for scalar variables, and Mantel tests related Bray–Curtis community dissimilarity to Euclidean distances of *z*-standardized environmental variables using 4999 permutations. An exploratory covariance-based structural equation model was fitted in lavaan [39] after *z*-standardization. Prespecified paths were PCN → Shannon diversity, pH → PCoA1, PCN → PCoA1, TOC → absolute *phoD* abundance, PCoA1 → AKP, absolute *phoD* abundance → AKP, AKP → Olsen-P, and TOC → Olsen-P. Maximum-likelihood estimation and 5000 bias-corrected bootstrap resamples were used. Fit was evaluated using the model *χ*^2^ test, comparative fit index, and standardized root-mean-square residual. All tests were two-sided; exact *P*-values are reported where possible. Soil Cu and Zn treatment means and FAVA values were presented descriptively unless supported by a corresponding inferential test.

## Results

### Mineral N intensification erodes the phoD-harbouring bacterial guild

**Figure 1:**
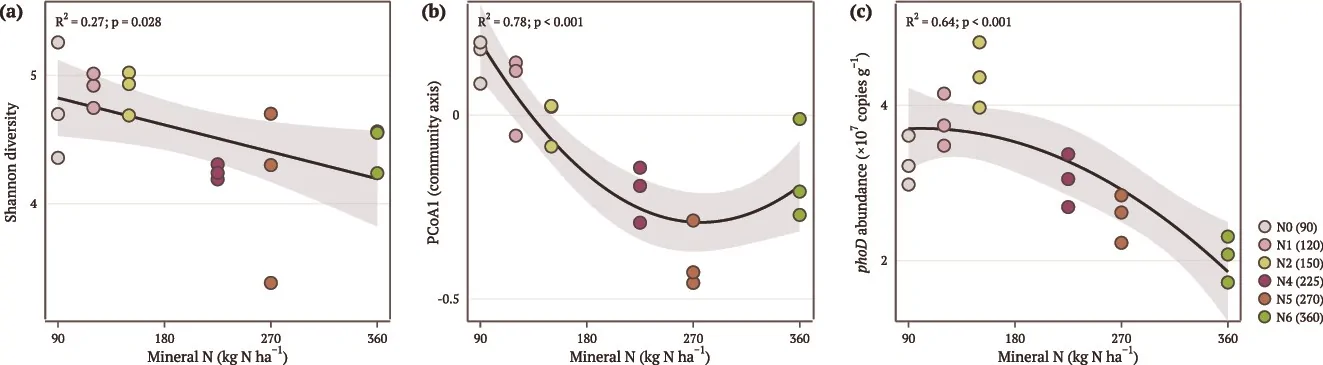
Responses of the *phoD*-harbouring bacterial guild to increasing mineral N input. (a) Shannon diversity, (b) the first principal-coordinate axis (PCoA1) of Bray–Curtis community dissimilarity, and (c) absolute *phoD* gene abundance measured by qPCR across six mineral N rates. Each point represents one biological replicate (*n* = 3 per treatment), and colours identify treatments. Solid lines represent a fitted linear regression in (a) and quadratic regressions in (b, c); grey bands indicate 95% confidence intervals. Model *R*^2^ and *P*-values are shown in the panels.

Across the mineral N dose gradient, Shannon diversity declined with increasing mineral N input (linear regression: *R*^2^ = 0.27, *P* = .028; Fig. 1a). PCoA1 scores followed a nonlinear relationship with mineral N rate, reaching their highest values near N4 (225 kg N ha^−1^ season^−1^) before declining at N5 and N6 (270–360 kg N ha^−1^ season^−1^; quadratic regression: *R*^2^ = 0.78, *P* < .001; Fig. 1b). Absolute *phoD* gene abundance showed a similar nonlinear response and was lowest at the highest N rates (quadratic regression: *R*^2^ = 0.64, *P* < .001; Fig. 1c). Thus, the highest mineral N inputs were associated with lower *phoD* diversity and gene abundance and with a directional shift in community composition.

### Manure substitution ameliorates soil acidity and increases C, N, and P status

**Figure 2:**
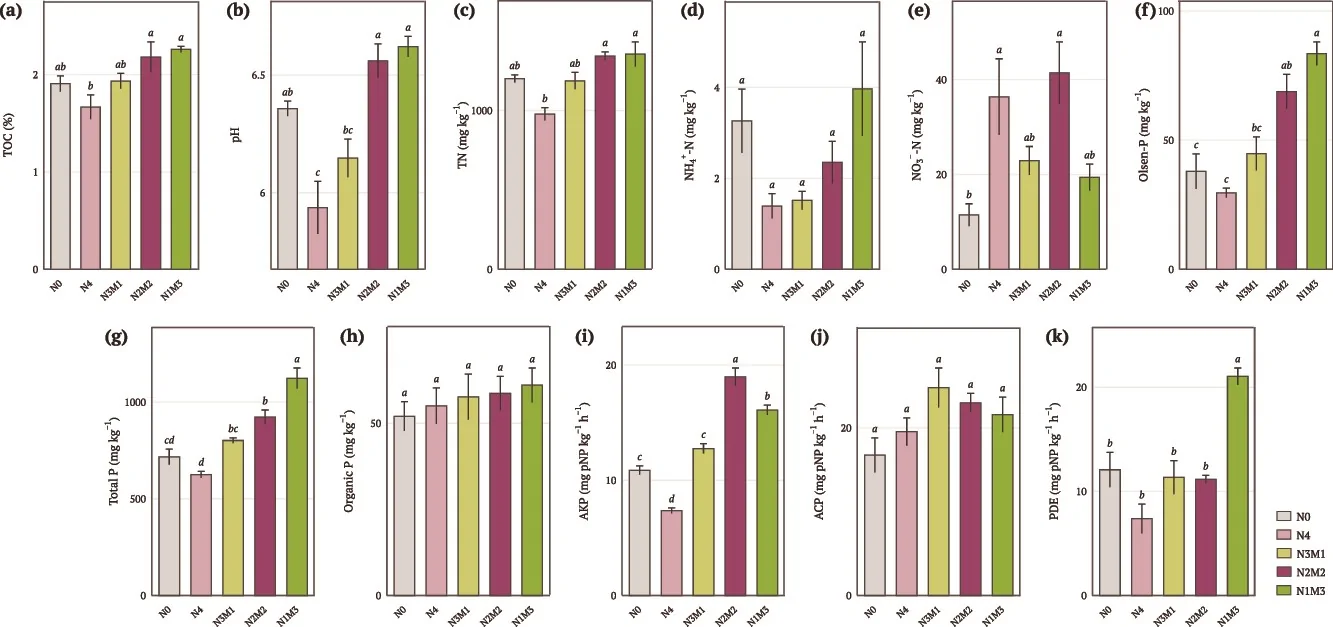
Soil biogeochemical properties and phosphatase activities across the ordered manure-substitution series. (a) Total organic carbon (TOC), (b) pH, (c) total nitrogen (TN), (d) ammonium nitrogen (NH_4_^+^–N), (e) nitrate nitrogen (NO_3_^−^–N), (f) Olsen-extractable phosphorus (Olsen-P), (g) total phosphorus (TP), (h) organic phosphorus (P_o_), (i) alkaline phosphatase activity (AKP), (j) acid phosphatase activity (ACP), and (k) phosphodiesterase activity (PDE). N0 is shown as the lowest mineral N baseline; N4, N3M1, N2M2, and N1M3 form the ordered manure-substitution series. Bars show means ± standard error (*n* = 3), except for P_o_ in N4 (*n* = 2 after one analytically impossible negative value was treated as missing). Treatments not sharing a lowercase letter differ at *P* < .05 within a variable.

Relative to the mineral-fertilizer reference N4, TOC was ~31% higher in N2M2 and 36% higher in N1M3 (Fig. 2a; Table S2). Soil pH increased from 5.94 in N4 to 6.15, 6.56, and 6.62 in N3M1, N2M2, and N1M3, respectively (Fig. 2b), while TN increased by 21%–39% (Fig. 2c). NH_4_^+^–N was 69% and 186% higher in N2M2 and N1M3 than in N4 (Fig. 2d), whereas NO_3_^−^–N was 47% lower in N1M3 (Fig. 2e).

Olsen-P and TP increased by 51%–182% and 28%–80%, respectively, across the manure-substituted treatments (Fig. 2f and g), whereas P_o_ did not differ significantly (Fig. 2h). AKP activity was 73%, 158%, and 119% higher in N3M1, N2M2, and N1M3, respectively, than in N4 and had its highest observed mean in N2M2 (Fig. 2i). ACP showed no significant treatment response (Fig. 2j), whereas PDE was 54% and 185% higher in N3M1 and N1M3, respectively (Fig. 2k). The ordered treatment series was therefore associated with reduced soil acidity and higher C, N, and P status and P-cycling enzyme activities.

### Manure substitution expands and restructures the phoD guild

**Figure 3:**
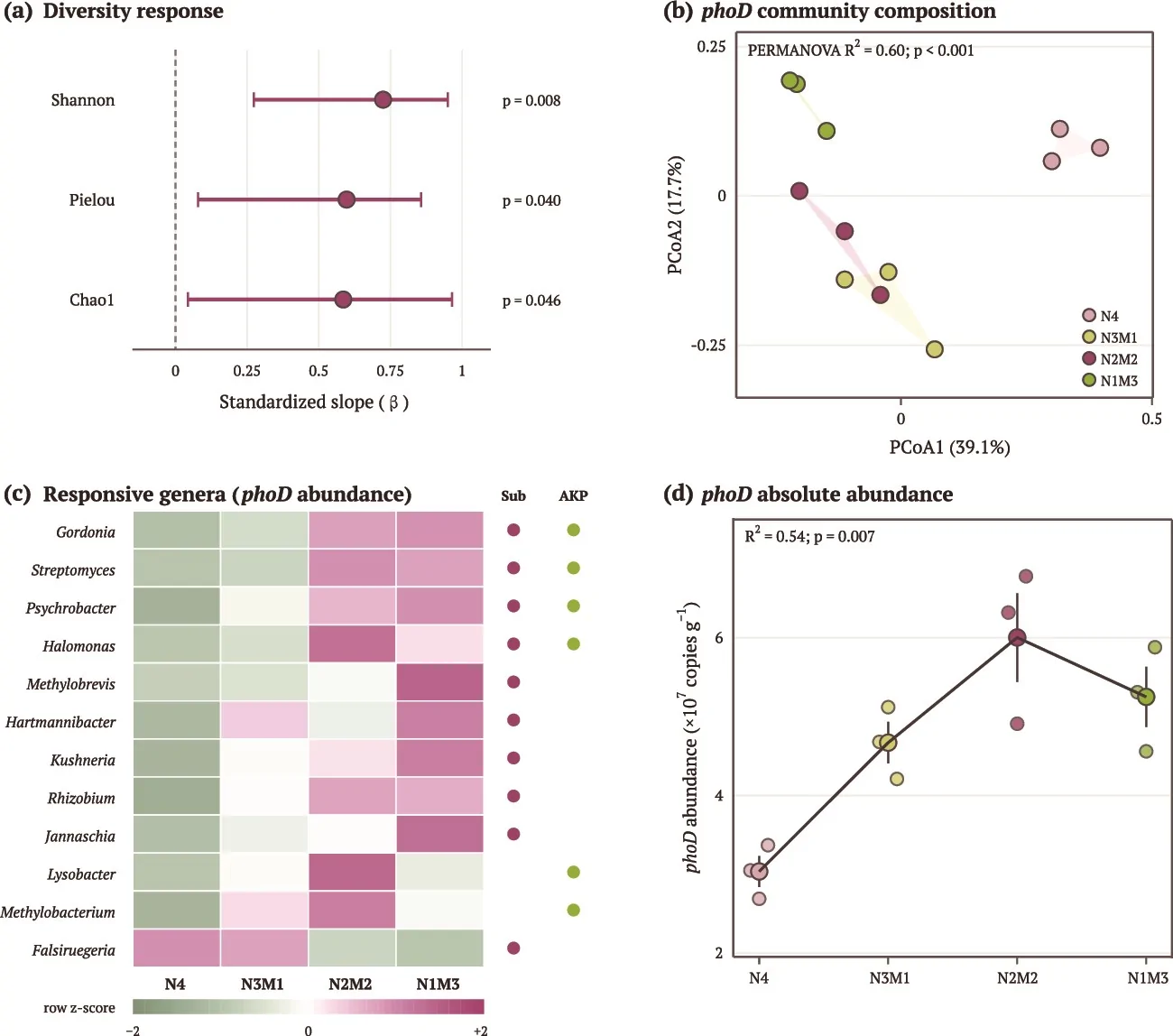
Manure substitution increases diversity and abundance and restructures the *phoD*-harbouring bacterial community. (a) Standardized slopes relating the ordered treatment score (N4 = 0, N3M1 = 1, N2M2 = 2, and N1M3 = 3) to Shannon diversity, Pielou’s evenness, and Chao1 richness. Points are standardized regression coefficients and horizontal lines are bootstrap 95% confidence intervals. (b) Principal coordinates analysis of Bray–Curtis community dissimilarities. Points represent biological replicates, coloured polygons delimit treatment-specific convex hulls, and the PERMANOVA result is shown. (c) Row-standardized heat map of qPCR-scaled putative genus-level *phoD* indices. Magenta circles indicate genera associated with the ordered treatment score, and green circles indicate genera associated with AKP after false-discovery-rate correction (*q* < 0.05). (d) Absolute *phoD* gene abundance. Small points represent biological replicates; large points and error bars show means ± standard error. The regression used replicate-level observations.

Shannon diversity, Pielou’s evenness, and Chao1 richness increased with the ordered manure-substitution score, with positive standardized slopes for all three indices (*P* = .008, .040, and .046, respectively; Fig. 3a). Community composition differed among N4, N3M1, N2M2, and N1M3 (PERMANOVA: *R*^2^ = 0.60, *P* < .001; Fig. 3b), with the strongest separation between N4 and N1M3 along PCoA1.

FAVA values were descriptively four- to eight-fold higher in N4 than in the manure-substituted treatments (Fig. S1). However, the corresponding PERMDISP test was not significant (*F* = 2.32, *P* = .131). The lower FAVA values therefore indicate a descriptive tendency towards lower among-replicate compositional variability rather than confirmatory evidence of convergence or a change in community-assembly mechanism.

Genus-level analyses identified ten putatively assigned *phoD* genera associated with the ordered treatment score after false-discovery-rate correction, including *Gordonia, Streptomyces, Psychrobacter*, and *Halomonas* (*q* < 0.05; Fig. 3c). These four genera were also associated with AKP activity. Assignments represent closest reference-based affiliations rather than definitive organismal identifications.

Absolute *phoD* abundance was positively associated with the ordered manure-substitution score (*R*^2^ = 0.54, *P* = .007; Fig. 3d). The treatment mean was highest in N2M2 and slightly lower in N1M3, but this descriptive maximum was not interpreted as a statistically identified breakpoint or optimum. Across replicate samples, absolute *phoD* abundance was positively associated with AKP activity (*R*^2^ = 0.78, *P* < .001; Fig. S2).

### Environmental filtering and microbial attributes jointly predict AKP and Olsen-P

**Figure 4:**
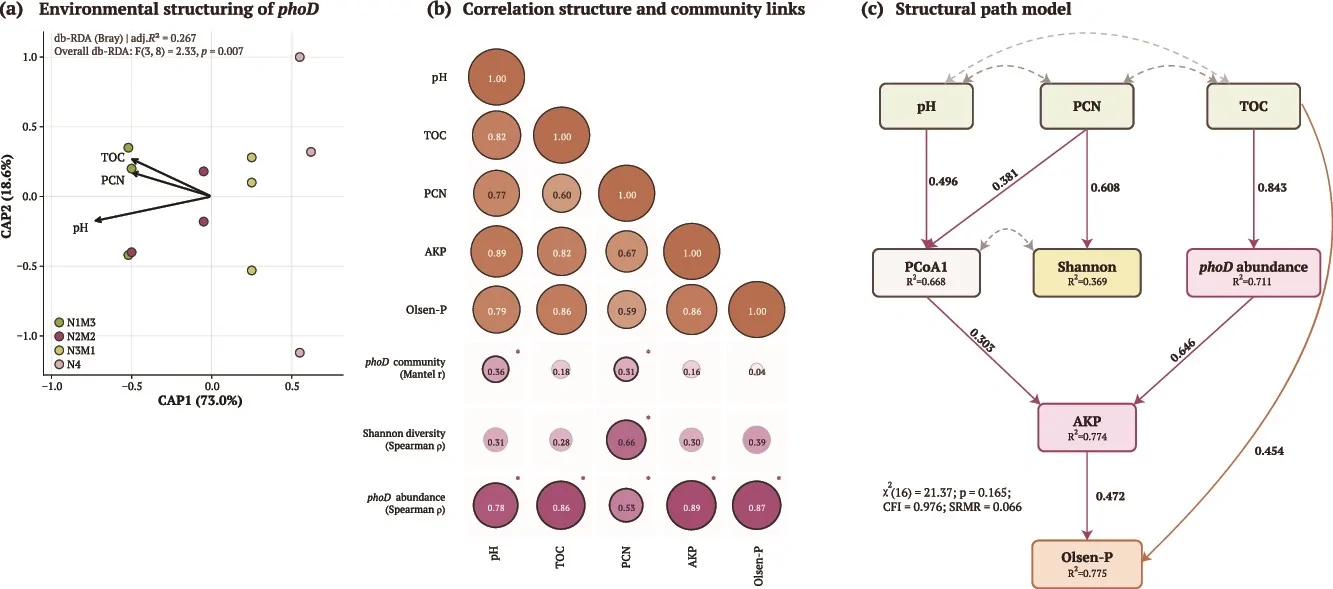
Environmental correlates and structural pathways linking the *phoD*-harbouring bacterial community to alkaline phosphatase activity and Olsen-P. (a) Distance-based redundancy analysis of Bray–Curtis community dissimilarities for N4, N3M1, N2M2, and N1M3 (*n* = 12), constrained by soil pH, TOC, and the principal-component soil N index (PCN). (b) Correlation structure across all ten treatments (*n* = 30). The upper matrix presents Spearman correlations among pH, TOC, PCN, AKP, and Olsen-P; lower rows show Mantel correlations for Bray–Curtis community dissimilarity and Spearman correlations for Shannon diversity and absolute *phoD* abundance. Circle size and colour intensity represent absolute effect size; asterisks indicate false-discovery-rate-adjusted *q* < 0.05. (c) Exploratory structural equation model fitted to all 30 samples. Single-headed arrows are prespecified directed paths, dashed double-headed arcs are estimated covariances, coefficients are standardized, and *R*^2^ values are shown for endogenous variables.

The soil predictors were correlated, with variance inflation factors ranging from 3.2 to 4.8 (Fig. S3). The distance-based redundancy model containing pH, TOC, and PCN explained a significant fraction of variation in *phoD* community composition within the manure-substitution series (*F*_3,8_ = 2.33, *P* = .007, adjusted *R*^2^ = 0.267; Fig. 4a). In marginal tests, pH retained an independently detectable association (*F* = 2.348, *P* = .046, partial *R*^2^ = 0.082), whereas TOC (*P* = .422) and PCN (*P* = .571) did not (Table S3). Given predictor correlation, pH is interpreted as the strongest measured marginal correlate rather than an experimentally isolated causal driver.

Across the integrated sample set, pH, TOC, and absolute *phoD* abundance were positively associated with AKP and Olsen-P, while community composition was associated with pH and PCN after false-discovery-rate correction (Fig. 4b). Within the manure-substitution series, PCoA1 remained associated with AKP after accounting for pH, TOC, and PCN (partial *R*^2^ = 0.70, *P* = .005; Fig. S4a), and AKP was positively associated with Olsen-P (*R*^2^ = 0.67, *P* = .001; Fig. S4b).

Variation partitioning accounted for 77% of the observed variation in AKP (Fig. S5). The largest unique fraction was associated with pH (0.094), whereas the shared fraction among pH, TOC, and TP was substantially larger (0.512). The predominance of shared variation indicated that the individual contributions of pH, C status, and P status could not be cleanly separated within this treatment series.

The exploratory structural equation model fitted to all 30 samples showed acceptable overall fit (*χ*^2^_16_ = 21.37, *P* = .165; comparative fit index = 0.976; standardized root-mean-square residual = 0.066; Fig. 4c). Soil pH and PCN were conditionally associated with PCoA1 (standardized *β* = 0.50 and 0.38), while PCN was associated with Shannon diversity (*β* = 0.61). TOC was associated with absolute *phoD* abundance (*β* = 0.84). PCoA1 and *phoD* abundance retained positive conditional associations with AKP (*β* = 0.30 and 0.65), whereas AKP and TOC were associated with Olsen-P (*β* = 0.47 and 0.45). The model was consistent with joint environmental and microbial contributions but did not demonstrate causal effects.

### Agronomic responses and contextual Cu/Zn accumulation

Within the four manure-substitution treatments, N2M2 had the highest observed mean rice grain yield and grain P removal, followed by slightly lower means in N1M3 (Fig. S6a and b). Because these variables were derived from archived plot-level records, they were used to place the microbial and soil responses in an agronomic context rather than to identify an optimum manure rate.

Treatment-level mean soil Cu and Zn concentrations increased across N4, N3M1, N2M2, and N1M3 and were highest in N1M3 (Fig. S6c). All displayed means were below the applicable pH-specific risk-screening values in GB 15618–2018. However, replicate-level metal data and complete analytical quality-control records were unavailable; the pattern was therefore evaluated descriptively and indicates a potential long-term trade-off rather than a demonstrated contamination effect.

**Figure 5:**
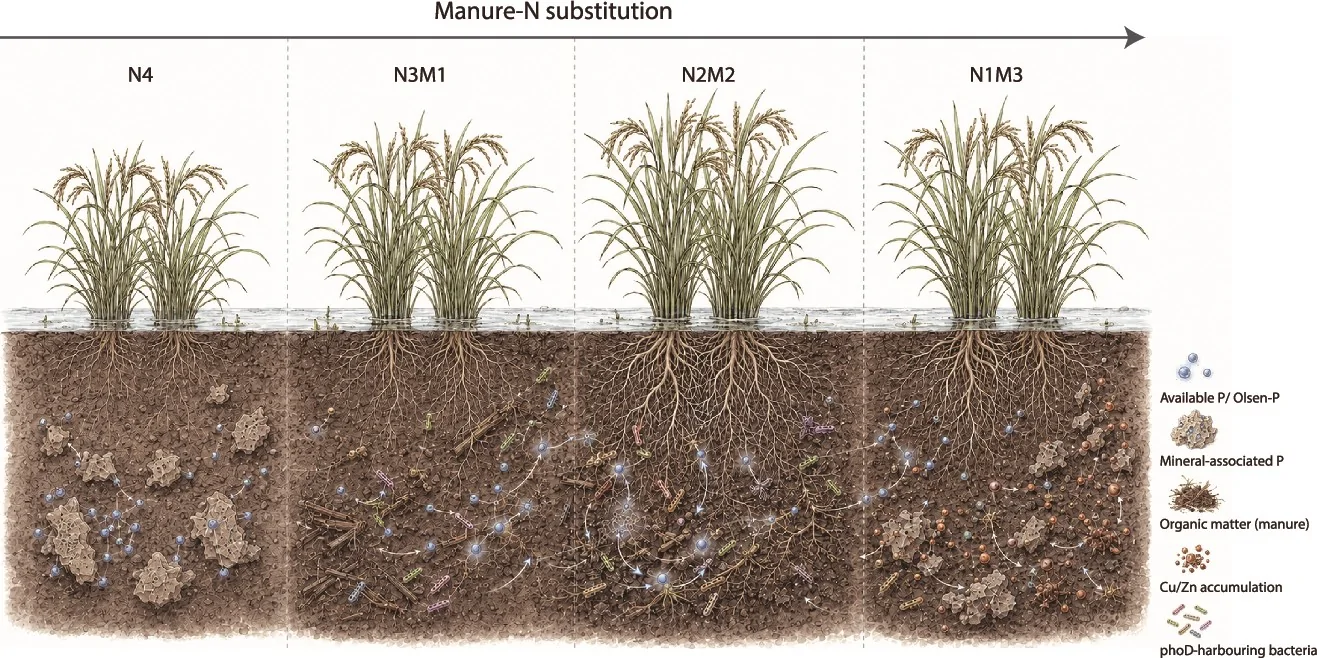
Conceptual synthesis of observed responses across the ordered manure-substitution series. The four panels represent N4, N3M1, N2M2, and N1M3, respectively. Increasing manure input is depicted together with the observed changes in soil properties, the *phoD*-harbouring bacterial community, potential alkaline phosphatase activity, Olsen-P, crop responses, and treatment-level Cu and Zn means. N2M2 had the highest observed means for several microbial and agronomic variables, whereas N1M3 retained high Olsen-P and had the highest Cu and Zn means. Spheres denote an operationally available P pool, aggregates denote an unspecified mineral-associated P, fragments denote manure-derived organic matter, rod-shape symbols denote putative *phoD*-harbouring bacteria, illuminated particles and arrows denote the hypothesized mobilization and plant acquisition of orthophosphate, and metal particles denote Cu/Zn accumulation. The symbols are qualitative, are not proportional to measured concentrations, and should not be interpreted as direct evidence of mineral-P dissolution or P flux.

Taken together, N2M2 showed the most favourable observed combination of *phoD* abundance, AKP activity, grain yield, and grain P removal among the four tested treatments, whereas N1M3 maintained higher Olsen-P than N4 but had the highest Cu and Zn means (Fig. 5). These observations motivate a management hypothesis to be tested with replicated nutrient budgets and metal measurements; they do not establish a universal agronomic optimum.

## Discussion

The two fertilization contrasts revealed opposing trajectories in the *phoD*-harbouring bacterial guild. Increasing mineral N input was associated with lower diversity and absolute gene abundance and a nonlinear shift in community composition, whereas increasing manure input accompanied by decreasing mineral N input was associated with higher soil pH, C and P status, potential AKP activity, and a larger and compositionally distinct *phoD* guild. Because the manure series altered several soil properties simultaneously, these responses should be interpreted as an integrated treatment effect rather than as evidence that N source alone controlled microbial P mobilization.

### Contrasting responses of the phoD guild to mineral N and manure

The decline in *phoD* diversity and abundance towards the highest mineral N rates is consistent with evidence that chronic N enrichment can acidify agricultural soils and reduce bacterial diversity and P-cycling functional potential [5, 12, 40, 41]. The nonlinear compositional response further indicates that the guild did not respond as a simple linear function of fertilizer dose. This distinction matters because community composition and gene abundance describe different aspects of functional potential: relative profiles identify taxonomic reorganization, whereas qPCR-based abundance describes changes in the size of the genetic reservoir [15]. Conversely, the ordered manure-substitution series supported both dimensions of the guild, consistent with long-term studies in which organic fertilization enriched active *phoD*-harbouring populations and strengthened links between these communities and phosphatase activity [14, 16, 17].

### Coupled edaphic filtering rather than an isolated pH effect

Soil pH was the only predictor with a detectable marginal association with community composition in the distance-based redundancy analysis, supporting its role as a major filter of bacterial distributions and nutrient-cycling functions [42, 43]. However, pH, TOC, PCN, and P pools changed together, and the variation-partitioning analysis assigned much more AKP variation to their shared fraction than to any unique fraction. This prevents a causal division in which pH alone determines composition and C alone determines abundance. Rather, manure may buffer acidity while simultaneously adding organic substrates and nutrients, as expected from broader evidence on manure-mediated soil C accumulation and acid-soil amelioration [23, 24]. The positive association between TOC and *phoD* abundance is therefore best viewed as a conditional relationship within this coupled gradient, not as proof of direct C limitation. This cautious interpretation is also consistent with recent syntheses emphasizing tight microbial C–P coupling and context-dependent nutrient allocation [9, 19].

### Community restructuring is associated with potential P mobilization

Both the principal community axis and absolute *phoD* abundance retained conditional associations with potential AKP activity, and AKP was associated with Olsen-P. Together, these results suggest that community reorganization and the size of the *phoD* reservoir may provide complementary information about P-cycling potential. Similar structure–function relationships have been reported where manure or long-term fertilization altered *phoD* communities together with alkaline phosphatase activity and P availability [11, 14, 18]. Nevertheless, DNA abundance is not gene expression, the enzyme assay measures potential activity under standardized laboratory conditions, and Olsen-P is an operational availability index rather than a direct P flux. The pathway from community change to P mobilization should therefore be regarded as a model-consistent interpretation requiring confirmation by transcript measurements and isotope-resolved P fluxes.

### Agronomic context, metal trade-offs, and limitations

N2M2 had the highest observed means for *phoD* abundance, AKP activity, grain yield, and grain P removal, but the four treatment levels do not define a statistically optimized manure rate. The pattern is compatible with agronomic studies reporting benefits of partial manure substitution and diminishing returns as manure input rises [25], but it cannot establish why the N2M2 means were highest. In particular, the proposed idea that microbial investment in P acquisition declines after P limitation is relieved remains a hypothesis because microbial P limitation was not measured directly. At the same time, soil Cu and Zn means increased towards N1M3. Long-term swine-manure experiments and recent reviews show that trace-metal loading can become an important constraint on otherwise beneficial organic amendments [26, 27, 44]. Although the displayed means remained below applicable risk-screening values, the absence of replicate-level metal measurements means that no treatment effect or accumulation rate can be inferred.

The study has further limitations. Three replicate plots sampled at one time point describe differences among long-term treatment states rather than temporal stability or trajectories. The predictors were correlated, so regression and structural-equation paths are conditional associations rather than causal effects. DNA-based sequencing and qPCR quantify genetic composition and reservoir size, not transcription or realized mineralization, and genus assignments based on a short functional-gene fragment are putative. Future tests should combine seasonal sampling, reverse-transcription qPCR or metatranscriptomics, isotope-based P flux measurements, complete plant P budgets, and replicated metal measurements. Such work would directly test whether the observed treatment contrasts reflect changes in microbial P-acquisition investment as proposed by current microbial P-cycling theory [10].

## Conclusion

After 10 years of contrasting fertilization, high mineral N input and increasing manure input were associated with opposing changes in the *phoD*-harbouring bacterial guild. The manure-substitution series combined higher soil pH, C and P status, *phoD* diversity and abundance, potential AKP activity, and Olsen-P, whereas the highest mineral N rates were associated with lower diversity and abundance. Multivariate analyses were consistent with coupled edaphic and microbial contributions to AKP and Olsen-P but did not isolate a single causal driver. Among the four manure-series treatments, N2M2 had the highest observed means for several microbial and agronomic responses, while Cu and Zn means increased towards N1M3. These results support manure substitution as a strategy for counteracting mineral N-associated deterioration of microbial P-cycling potential in acidified paddy soil, while underscoring the need to verify nutrient balances, P fluxes, and long-term metal accumulation across sites and seasons.

## Supplementary Material

Supplementary_material_ycag261

## Data Availability

Raw *phoD* amplicon sequences have been deposited in the NCBI Sequence Read Archive under BioProject PRJNA1357597; associated BioSample and Sequence Read Archive run accessions (SRR35948752–SRR35948781) are listed in Table S4. The curated *phoD* protein reference database (Data S1: phoD_reference.faa) and the ASV feature and taxonomy tables (Data S2: phoD_feature_taxonomy.zip, containing feature-table.tsv and taxonomy.tsv) are provided as Supplementary Data files.
