# Supplementary material for: Manure substitution counteracts mineral nitrogen-driven erosion of the phoD guild and supports phosphorus mobilization in acidified paddy soil
Source: ISME Commun. 2026 Sep 10;6(1):ycag261. doi: 10.1093/ismeco/ycag261 (PMC13615535; doi:10.1093/ismeco/ycag261)
Supplement: Supplementary file 1 [file supplementary_material_ycag261.zip › 2. SI-final.pdf]

Supplementary Information for

Manure substitution counteracts mineral nitrogen-driven erosion of the *phoD* guild and sustains phosphorus mobilisation in acidified paddy soil

Jitao Huang[1,2#], Bangxiao Zheng[1,3,4,5,#*], Yuxuan Mo[4,6], Salimzoda Amonullo[7], Weiyi Lv[1], Xianyong Lin[8], Qing-Fang Bi[9,10,11**]

[1] *Center for Ecology & Health Innovative Research, Xiamen University of Technology, Xiamen 361024, P. R. China*;

[2] *Fenner School of Environment & Society, The Australian National University, Canberra, ACT 2601, Australia;*

[3] *Xiamen Key Laboratory of Membrane Research and Application, Xiamen 361024, P. R. China;*

[4] *CSIC, Global Ecology Unit CREAF-CEAB-UAB, Bellaterra 08193, Spain;*

[5] *Faculty of Biological and Environmental Sciences, Ecosystems and Environment Research Programme, Niemenkatu 73, FI-15140, Lahti, University of Helsinki, Finland;*

[6] *Yunnan Key Laboratory of Forest Ecosystem Stability and Global Change, Xishuangbanna Tropical Botanical Garden, Chinese Academy of Sciences, Mengla, Yunnan 666303, P. R. China;*

[7] *Institute of Soil Sciences and Agrochemistry, Tajik Academy of Agricultural Sciences, Dushanbe, 734025, Tajikistan;*

[8] *MOE Key Laboratory of Environment Remediation and Ecological Health, College of Environmental and Resource Sciences, Zhejiang University, Hangzhou 310058, PR China;*

[9] *Department Biogeochemical Integration, Max Planck Institute for Biogeochemistry, 07745, Jena, Germany;*

[10] *Institute of Agricultural and Nutritional Sciences, Soil Biogeochemistry, Martin Luther University Halle-Wittenberg, 06120, Halle (Saale), Germany;*

[11] *German Centre for Integrative Biodiversity Research (iDiv) Halle-Jena-Leipzig, D-04103 Leipzig, Germany.*

[#] These authors contributed to this study equally.

Corresponding Author:

[*] Bangxiao Zheng, Email: bangxiao.zheng@xmut.edu.cn

[**] Qingfang Bi, Email: qbi@bgc-jena.mpg.de

Supplementary Methods

Reference-based taxonomic annotation of phoD ASVs

Reference *phoD*-encoded protein sequences were retrieved from UniProtKB using the query gene_exact:phoD AND taxonomy_id:2 (downloaded on 22 September 2025; UniProt release 2025_02). Records annotated as fragments or partial sequences were excluded, and proteins between 350 and 800 amino acids in length were retained to reduce the inclusion of incomplete or anomalously short sequences. The resulting curated protein dataset was used as the reference database for annotation and is provided as **Data S1** (*phoD_reference.faa*).

Representative *phoD* ASV nucleotide sequences were compared against the curated protein reference database using FrameBot v1.1 implemented in RDP Tools. Analyses were conducted using bacterial translation table 11, a minimum protein-sequence identity threshold of 40%, and the BLOSUM62 substitution matrix. FrameBot translated each nucleotide sequence, detected and corrected potential frameshift errors, and aligned the translated sequence against the reference proteins. For each ASV, the highest-scoring reference match satisfying the identity threshold was retained, together with its UniProt accession and alignment statistics. ASVs without a qualifying reference match were recorded as unclassified.

Taxonomic labels were derived from the organism annotation associated with each best-matching UniProt accession. Because this reference-based procedure does not provide sufficient resolution for reliable species-level identification, assignments were restricted to the putative genus level. The genus name was extracted from the UniProt organism field and formatted as k__Bacteria; g__Genus; records for which a valid genus name could not be resolved were designated k__Bacteria; g__Unclassified. The resulting assignments were compiled into a QIIME 2-compatible taxonomy table and linked to the ASV feature table for genus-level community analyses. These assignments should therefore be interpreted as the closest reference-based affiliations of the amplified *phoD* sequences rather than definitive organismal identifications.

Sequence processing and downstream community analyses were performed in a Windows 10 environment using FrameBot v1.1, QIIME 2 version 2024.2, and OpenJDK 11.0.27. The curated reference protein database is provided as **Data S1**, whereas the ASV feature table and corresponding taxonomy table are provided as **Data S2**.

Table S1. Loadings and sample scores for the principal-component soil N index (PCN).

S1a. PCN loadings and variance explained.

Axis	Variance explained (%)	Variable	Loading
		TN	0.690
PCN	`51.53	NH_4^+	0.673
		NO_3^-	−0.267

S1b. Sample-level PCN scores used in the integrated analyses.

Sample ID	Treatment	PCN score
N0-1	N0	0.005
N0-2	N0	1.660
N0-3	N0	0.741
N1-1	N1	0.307
N1-2	N1	1.151
N1-3	N1	-0.039
N2-1	N2	-0.108
N2-2	N2	0.738
N2-3	N2	-0.490
N4-1	N4	-1.474
N4-2	N4	-2.009
N4-3	N4	-1.031
N5-1	N5	-1.435
N5-2	N5	-0.923
N5-3	N5	-0.956
N6-1	N6	-1.723
N6-2	N6	-0.799
N6-3	N6	-1.844
N3M1-1	N3M1	-0.403
N3M1-2	N3M1	-0.043
N3M1-3	N3M1	-0.587

Sample ID	Treatment	PCN score
N2M2-1	N2M2	0.440
N2M2-2	N2M2	0.369
N2M2-3	N2M2	0.018
N1M3-1	N1M3	2.291
N1M3-2	N1M3	0.627
N1M3-3	N1M3	1.803
N4M2-1	N4M2	0.813
N4M2-2	N4M2	-1.247
N4M2-3	N4M2	-0.837

Notes: PCN was fitted once as the first principal component of z-standardized soil total N (TN), NH_4^+-N, and NO_3^--N in the 12 samples from the ordered manure-substitution series (N4, N3M1, N2M2 and N1M3). The axis was oriented so that higher PCN scores represented higher TN and NH_4^+-N but lower NO_3^--N. The fitted centre, scale, and loadings were then applied unchanged to project the remaining 18 samples. The same PCN scores should be used in all downstream db-RDA, correlation, and structural equation analyses.

Table S2. Soil biogeochemical properties and phosphatase activities in the low-mineral-N baseline and ordered manure-substitution treatments.

Variable	N0	N4	N3M1	N2M2	N1M3
**TOC**	1.91 ± 0.14 ab	1.67 ± 0.22 b	1.87 ± 0.10 ab	2.19 ± 0.27 a	2.27 ± 0.17 a
**pH**	6.36 ± 0.06 ab	5.94 ± 0.19 c	6.16 ± 0.14 bc	6.56 ± 0.32 a	6.62 ± 0.24 a
**TN**	1201.19 ± 41.46 bc	977.56 ± 69.89 d	1187.30 ± 90.63 c	1342.97 ± 46.56 ab	1354.41 ± 135.01 a
**NH_4^+**	3.26 ± 1.22 ab	1.39 ± 0.47 c	1.51 ± 0.35 c	2.35 ± 0.80 ab	3.97 ± 1.79 a
**NO_3^-**	11.47 ± 4.09 c	36.36 ± 13.91 ab	22.90 ± 5.19 bc	41.41 ± 11.34 a	19.38 ± 4.85 c
**Olsen-P**	37.91 ± 11.76 b	29.60 ± 3.12 b	44.82 ± 11.20 b	68.78 ± 11.56 a	83.51 ± 7.87 a
**TP**	716.56 ± 69.55 cd	625.57 ± 29.16 d	801.55 ± 24.47 c	922.05 ± 64.54 b	1123.02 ± 92.67 a
**Organic P**	52.01 ± 7.36 a	54.77 ± 5.24 a	57.65 ± 11.46 a	58.64 ± 8.63 a	61.06 ± 8.64 a
**AKP**	10.87 ± 0.66 cd	7.36 ± 1.52 d	12.76 ± 1.08 bc	18.97 ± 4.64 a	16.10 ± 2.23 ab
**ACP**	16.76 ± 3.56 b	19.55 ± 2.88 ab	24.80 ± 4.07 a	22.99 ± 1.95 ab	21.60 ± 3.61 ab
**PDE**	12.07 ± 2.90 b	7.37 ± 2.44 c	11.35 ± 2.78 b	11.15 ± 0.68 bc	21.03 ± 1.41 a

Notes: Values are means ± SD (n = 3), except for organic P in N4 (n = 2), for which one analytically impossible negative value was treated as missing before calculation. N0 is shown as the low-mineral-N baseline but is not part of the ordered manure-substitution series. Within each variable, treatments not sharing a lowercase letter differed significantly according to one-way ANOVA followed by Duncan's multiple-range test ($p < 0.05$). Residual normality and variance homogeneity were checked using Shapiro-Wilk and Levene tests, respectively. Units: TOC, %; TN, TP, Olsen-P, organic P, NH_4^+-N, and NO_3^--N, mg kg^{-1}; AKP, ACP, and PDE, mg pNP kg^{-1} h^{-1}. AKP, ACP, and PDE denote alkaline phosphatase, acid phosphatase, and phosphodiesterase activities, respectively.

Table S3. Marginal effects of soil predictors on the composition of the *phoD*-harbouring bacterial community in distance-based redundancy analysis.

**Variable**	**F (marginal)**	**p (marginal)**	**Partial R^2 (Δadj.R^2)**	**Significance**
pH	2.348	0.046	0.082	*
TOC	1.012	0.422	0.001	ns
PCN	0.788	0.571	−0.017	ns

Notes: The db-RDA was based on Bray-Curtis dissimilarities among the equal-N manure-substitution samples. Marginal permutation tests assessed the independent contribution of each predictor while retaining the remaining predictors in the model (9,999 permutations). Partial R^2 was calculated as the change in adjusted R^2 after removal of the corresponding predictor. Negative partial R^2 values indicate no detectable unique explanatory contribution and were retained rather than truncated to zero. *$p < 0.05$; ns, not significant.

Table S4. Sample metadata and NCBI BioSample and Sequence Read Archive accessions for the *phoD* amplicon dataset.

Sample ID	Treatment	BioSample accession	SRA Run accession
phoDN0-1	N0	SAMN53087279	SRR35948781
phoDN0-2	N0	SAMN53087280	SRR35948780
phoDN0-3	N0	SAMN53087281	SRR35948769
phoDN1M3-1	N1M3	SAMN53087285	SRR35948755
phoDN1M3-2	N1M3	SAMN53087286	SRR35948754
phoDN1M3-3	N1M3	SAMN53087287	SRR35948753
phoDN1-1	N1	SAMN53087282	SRR35948758
phoDN1-2	N1	SAMN53087283	SRR35948757
phoDN1-3	N1	SAMN53087284	SRR35948756
phoDN2M2-1	N2M2	SAMN53087291	SRR35948777
phoDN2M2-2	N2M2	SAMN53087292	SRR35948776
phoDN2M2-3	N2M2	SAMN53087293	SRR35948775
phoDN2-1	N2	SAMN53087288	SRR35948752
phoDN2-2	N2	SAMN53087289	SRR35948779
phoDN2-3	N2	SAMN53087290	SRR35948778
phoDN3M1-1	N3M1	SAMN53087294	SRR35948774
phoDN3M1-2	N3M1	SAMN53087295	SRR35948773
phoDN3M1-3	N3M1	SAMN53087296	SRR35948772
phoDN4M2-1	N4M2	SAMN53087300	SRR35948767
phoDN4M2-2	N4M2	SAMN53087301	SRR35948766
phoDN4M2-3	N4M2	SAMN53087302	SRR35948765
phoDN4-1	N4	SAMN53087297	SRR35948771
phoDN4-2	N4	SAMN53087298	SRR35948770
phoDN4-3	N4	SAMN53087299	SRR35948768
phoDN5-1	N5	SAMN53087303	SRR35948764
phoDN5-2	N5	SAMN53087304	SRR35948763

Sample ID	Treatment	BioSample accession	SRA Run accession
phoDN5-3	N5	SAMN53087305	SRR35948762
phoDN6-1	N6	SAMN53087306	SRR35948761
phoDN6-2	N6	SAMN53087307	SRR35948760
phoDN6-3	N6	SAMN53087308	SRR35948759

Notes: All sequence data were deposited in the NCBI Sequence Read Archive under BioProject PRJNA1357597. Sample identifiers correspond to those used in the feature table, environmental dataset, and downstream community analyses.

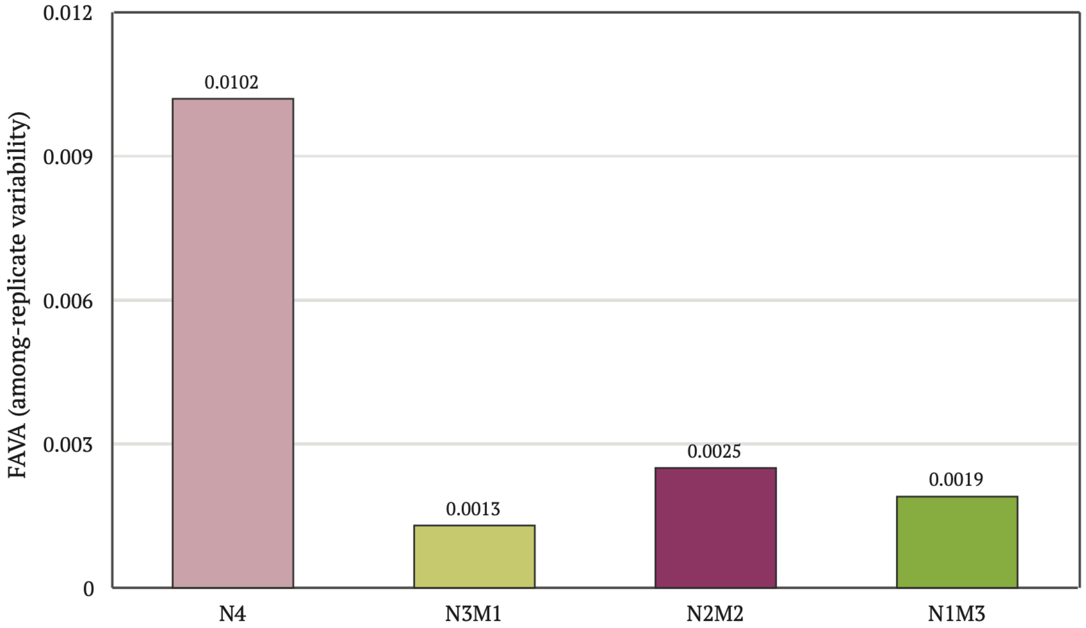

Figure S1. Among-replicate compositional variability of the *phoD*-harbouring bacterial community across the ordered manure-substitution series. Bars show the FAVA-based among-replicate variability index calculated within each treatment from three biological replicates. Lower values indicate greater compositional consistency among replicate communities. Values are presented descriptively and should not be interpreted as treatment means with independent replicate-level uncertainty.

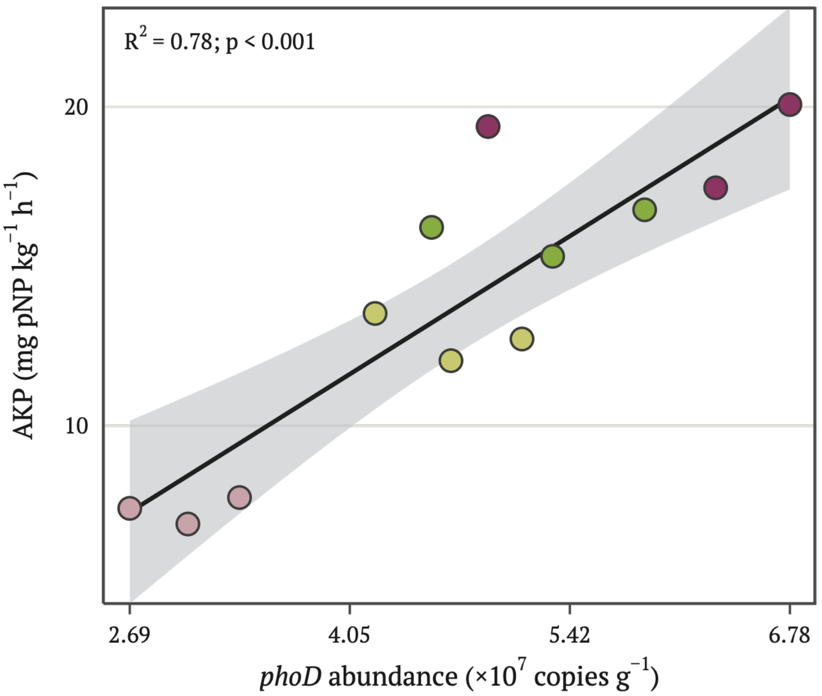

Figure S2. Relationship between absolute *phoD* gene abundance and alkaline phosphatase activity across the ordered manure-substitution series. Points represent biological replicates from N4, N3M1, N2M2, and N1M3. The solid line is the fitted linear regression and the grey band represents the 95% confidence interval. Model R^2 and p values are shown in the panel.

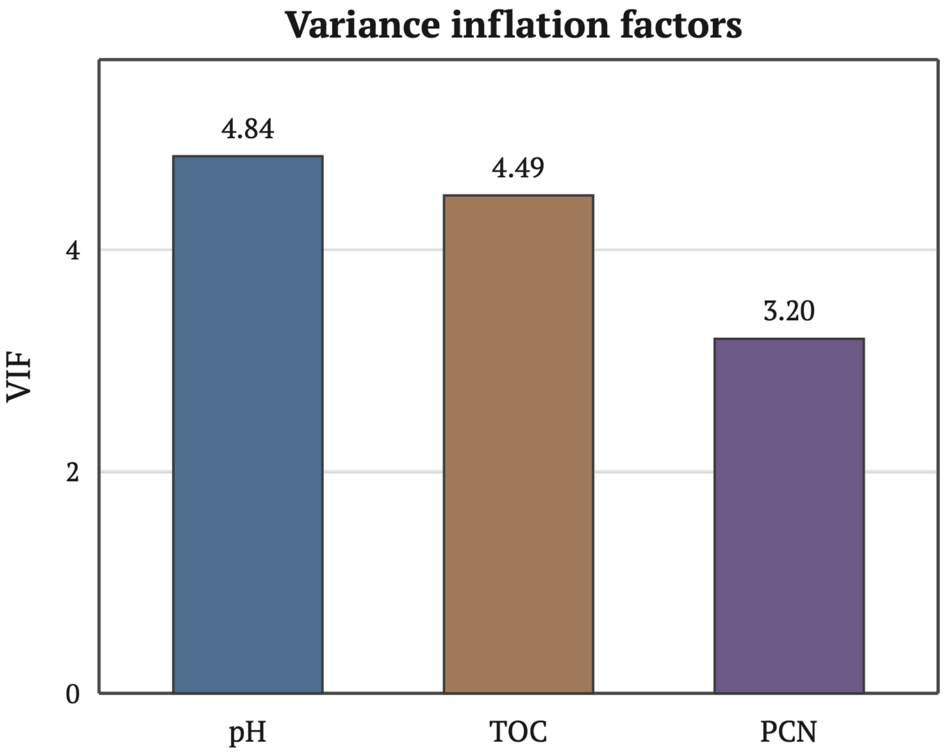

Figure S3. Variance inflation factors for the environmental predictors used in the integrated models. Variance inflation factors (VIFs) are shown for soil pH, total organic carbon (TOC), and the principal-component soil N index (PCN). Values above the bars are the corresponding VIF estimates.

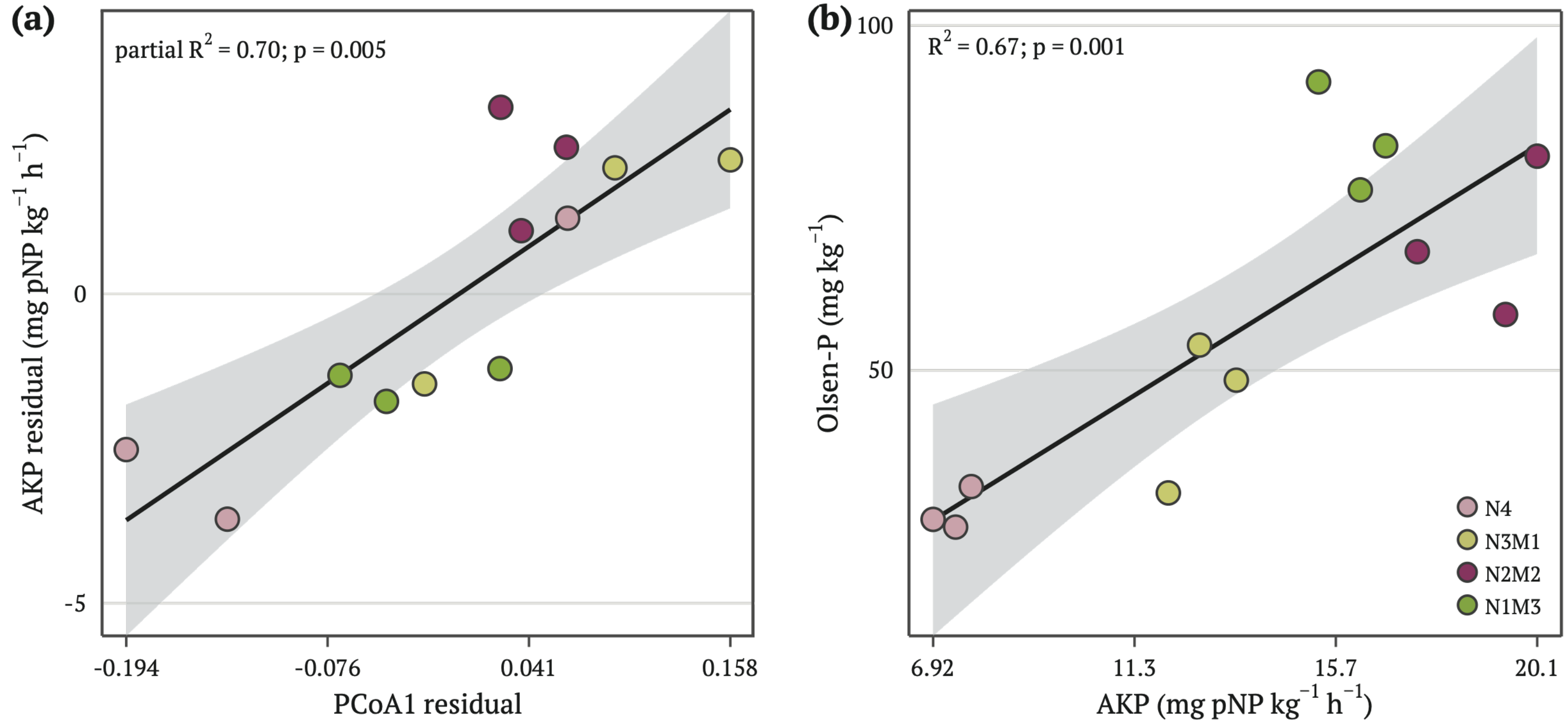

Figure S4. Relationships linking *phoD* community composition, alkaline phosphatase activity, and Olsen-P across the ordered manure-substitution series. (a) Partial-residual relationship between the first *phoD* community axis (PCoA1) and alkaline phosphatase activity (AKP) after controlling for soil pH, TOC, and PCN. (b) Bivariate relationship between AKP and Olsen-P. Points represent biological replicates, solid lines show fitted linear regressions, and grey bands represent 95% confidence intervals. Partial or ordinary R^2 and p values are shown in the respective panels.

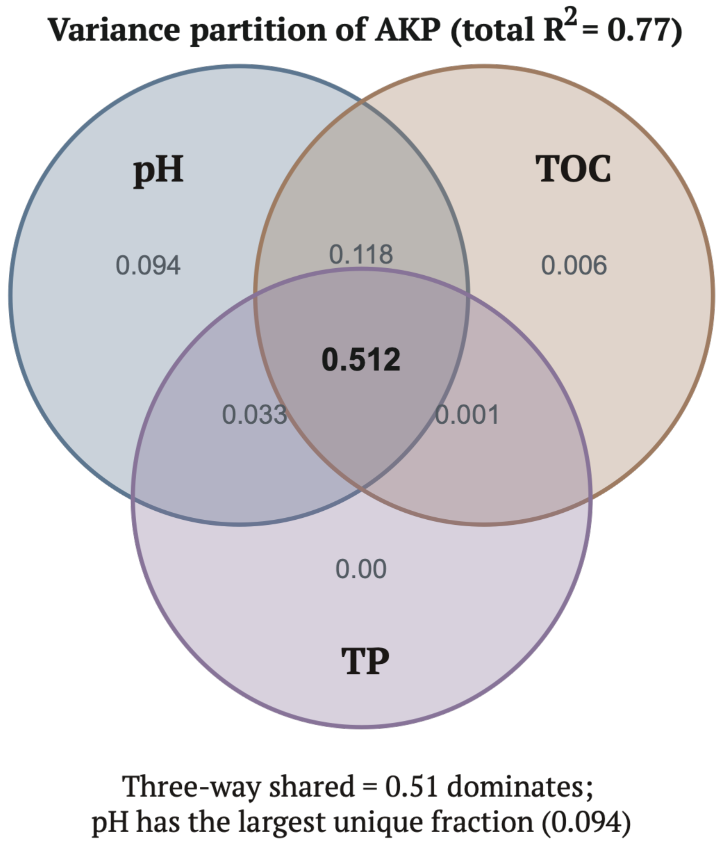

Figure S5. Variation partitioning of alkaline phosphatase activity among soil pH, total organic carbon, and total P. The Venn diagram partitions the variance in AKP explained uniquely and jointly by soil pH, total organic carbon (TOC), and total P (TP). Values are fractions of explained variance; the total model R^2 was 0.77. The central overlap represents variance jointly attributable to all three predictors, whereas the unexplained fraction is not displayed.

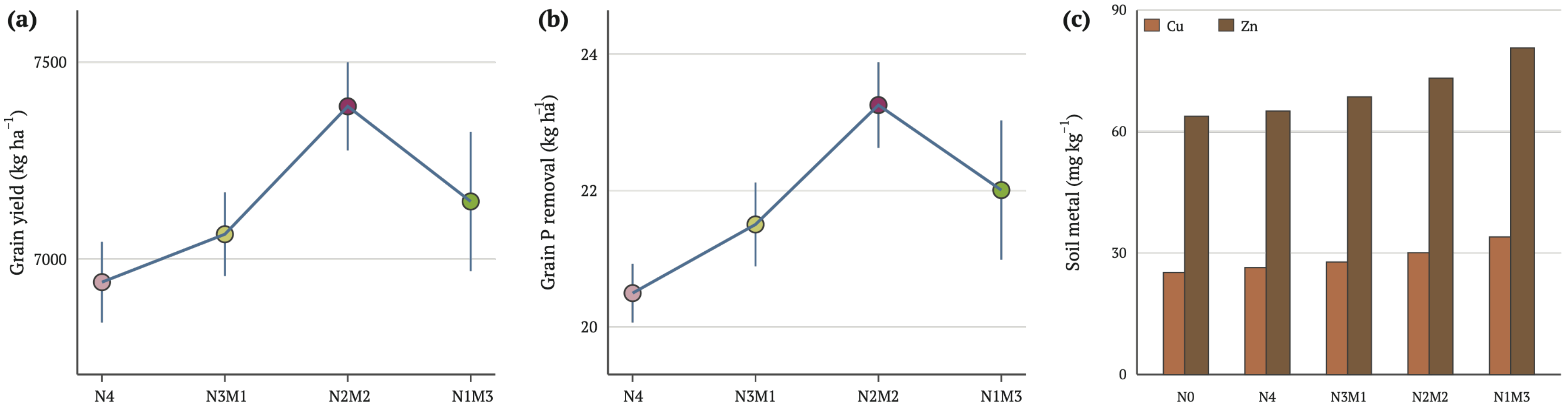

Figure S6. Agronomic responses and soil Cu/Zn accumulation across the ordered manure-substitution series. (a) Rice grain yield and (b) plant P uptake across N4, N3M1, N2M2 and N1M1 treatments. Points and error bars show means ± SE (n = 3), and lines connect treatment means. (c) Soil Cu and Zn concentrations in the low-mineral-N baseline treatment (N0) and the equal-N manure-substitution treatments. Cu and Zn values are treatment-level means and are presented descriptively because replicate-level measurements were unavailable.
